# Post-varicella vaccination uveitis in a child with nephrotic syndrome receiving immunosuppressive treatment: a case report

**DOI:** 10.3389/fped.2025.1567164

**Published:** 2025-04-16

**Authors:** Catarina Andrade, Miguel de Almeida Cordeiro, Rute Baeta Baptista, Beatriz Sousa Nunes, Ana Margarida Garcia, Tiago Milheiro Silva, Marta Valente Pinto

**Affiliations:** ^1^ Infectious Diseases Unit, Hospital Dona Estefânia, Unidade Local de Saúde São José, Lisbon, Portugal; ^2^ Pediatrics, Hospital Central do Funchal, SESARAM, EPERAM, Funchal, Portugal; ^3^ Ophthalmology, Unidade Local de Saúde de Lisboa Ocidental, Lisbon, Portugal; ^4^ Paediatric Nephrology Unit, Hospital Dona Estefânia, Unidade Local de Saúde São José, Lisbon, Portugal; ^5^ NOVA Medical School, Faculdade de Ciências Médicas, NMS, FCM, Universidade Nova de Lisboa, Lisboa, Portugal; ^6^ Centro Clínico Académico de Lisboa, Lisbon, Portugal; ^7^ Consulta Rastreio Infecioso e Imunossupressor Pré-Tratamento Imunossupressor, Hospital Dona Estefânia, Unidade Local de Saúde de São José, Lisbon, Portugal; ^8^ Primary Immunodeficiency Unit, Hospital Dona Estefânia, Unidade Local de Saúde São José, Lisbon, Portugal; ^9^ Egas Moniz Center for Interdisciplinary Research (CiiEM), Egas Moniz School of Health and Science, Caparica, Portugal

**Keywords:** attenuated vaccine, immunocompromised patient, nephrotic syndrome, uveitis, varicella vaccine

## Abstract

Patients with nephrotic syndrome are at heightened risk of infections due to the underlying disease pathophysiology and the effects of immunosuppressive therapies. Varicella-zoster virus (VZV) infection can cause severe complications in immunocompromised individuals. Concerns about the safety of live attenuated vaccines in this population persist. Emerging vaccination strategies incorporate pre-vaccination risk stratification algorithms based on immunological criteria. We present a case of a five-year-old male with corticosteroid-dependent nephrotic syndrome, in complete remission on mycophenolate mofetil therapy, who received the varicella vaccine after meeting immunocompetence criteria. Fourteen days post-vaccination, he developed scant vesicular lesions, with VZV DNA detected by PCR via swab. By day 16 post-vaccination, he presented with left-eye panuveitis. VZV DNA was also detected in the blood by PCR. Differentiation of VZV vaccine strains from wild-type strains was not possible. Additionally, molecular testing for VZV in the aqueous humor was not performed. However, given the temporal association with varicella vaccination, the detection of VZV in the blood and cutaneous lesions, and most importantly, the immunosuppression of the patient, post-vaccination ocular varicella was assumed even without an epidemiological history of varicella exposure. This case highlights the importance of a thorough immunocompetence assessment before administering live vaccines to immunosuppressed patients, as well as close post-vaccine monitoring and a high index of suspicion for complications to optimize vaccine safety in this vulnerable group. Patients with nephrotic syndrome require vaccination strategies tailored to their individual risk.

## Introduction

Nephrotic syndrome is characterized by nephrotic-range proteinuria, hypoalbuminemia, and/or edema. It is the most common glomerular disorder in children, with an annual incidence ranging from 1.15 to 16.9 per 100,000 children globally (1). Most children with nephrotic syndrome (85%–90%) will achieve complete remission within 4–6 weeks of steroid treatment and are classified as steroid-sensitive (2). Among those who are steroid-sensitive, 70%–80% will experience at least one relapse, and up to half may develop frequent relapses or become dependent on steroids to maintain remission. In such cases, steroid-sparing immunosuppressive drugs are often used to reduce long-term steroid exposure (2). Nephrotic syndrome presents an elevated risk of severe infections due to multifactorial immunosuppression, to which immunosuppressive treatment, hypogammaglobulinemia, and urinary loss of complement components contribute (3).

While varicella is generally regarded as a benign, self-limiting disease in children, both varicella and its complications tend to be more severe in immunocompromised individuals. These patients frequently exhibit extensive rashes, and are at risk of additional complications, including bacterial superinfection, pneumonia, hepatitis, and central nervous system involvement due to varicella-zoster virus (VZV) (4–6). Although this population represents only 0.1% of varicella cases, it accounted for up to 25% of varicella-related fatalities in the pre-vaccine era (7). Furthermore, varicella can trigger an exacerbation of the existing disease, such as the recurrence of nephrotic syndrome, either through the immune response to the infection or due to the need to reduce immunosuppressive therapy in the context of infection (8). Thus, immunization is essential to prevent varicella infection (9). The varicella vaccine is a live attenuated vaccine, consisting of the Oka strain (6). It is approved for use from 12 months of age. In Portugal, universal vaccination of immunocompetent children is not recommended. Current guidelines restrict pediatric vaccination to adolescents with no prior history of varicella and to children who are in regular contact with immunocompromised patients (10, 11).

The safety of live attenuated vaccines is particularly concerning because of the potential risk of developing infection from the vaccine strains, especially for immunocompromised hosts (3). When feasible, clinicians should administer the varicella vaccine in VZV-naïve patients before initiation of immunosuppressive drugs (11).

In the pharmacological immunization package insert, immunosuppressive therapy is generally listed as a contraindication (12). However, in 2012, the European Medicines Agency recommended that, although the varicella vaccine should be avoided in patients with severely compromised immune systems, it could be considered in cases of milder immunosuppression (13).

The challenge for clinicians lies in evaluating the safety and efficacy of vaccines in immunodeficient conditions, especially with emerging therapies (11). An increasing number of studies advocate for tailor-made vaccination approaches in immunosuppressed patients, using pre-vaccination stratification systems that prioritize immunological criteria to assess patient immunocompetence, rather than focusing solely on the type of immunosuppressive drug (14).

Ocular involvement can occur during primary varicella infection, herpes zoster reactivation, or following varicella vaccination (15). Immunocompromised children are at a higher risk of optic nerve and retinal damage (15).

**Figure 1:**
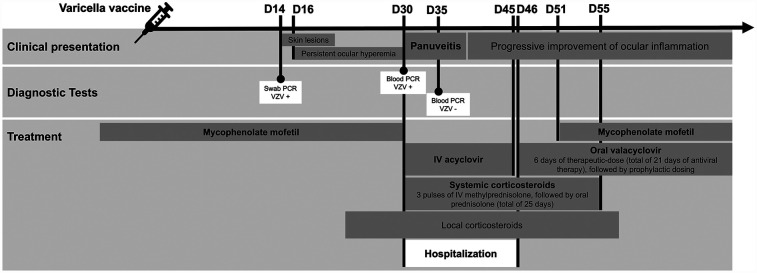
Timeline of the clinical case events. Chronology of the clinical case according to days following the administration of the varicella vaccine. IV, intravenous; PCR, polymerase chain reaction; VZV, varicella-zoster virus; +, positive; -, negative.

In this report, we describe a case of corticosteroid-dependent nephrotic syndrome, treated with mycophenolate mofetil (MMF), clinically stable for several months, that developed invasive disease following VZV vaccination. Figure 1 represents the timeline of the clinical case events. The legal representative of the child patient provided informed consent for this report.

## Case description

We present the case of a five-year-old male with a history of steroid-dependent nephrotic syndrome since the age of three. He achieved complete remission on corticosteroids and MMF treatment, followed by corticosteroid withdrawal and sustained remission on MMF monotherapy. During routine infectious screening, he had no documented history of varicella, and negative serologies for measles and varicella were noted. His parents expressed a desire to proceed with the corresponding vaccinations and provided informed consent after being informed of the risks.

It was decided to apply the Speth et al. (14) checklist to assess immunological capacity prior to vaccination. Regarding ongoing immunosuppressive therapy, prednisolone had been discontinued three months prior, and he was maintained solely on MMF (1,194 mg/m²/day). No family history or clinical evidence suggestive of primary immunodeficiency, nor any known hypersensitivity to the components of the varicella vaccine was documented. The patient received the mumps-rubella-measles (MMR) vaccination more than four weeks prior with no reported adverse events. Clinically, his underlying disease was stable, with no recent relapses, no therapy adjustments for at least three months, and no active infection. He met the immunocompetence requirements (Table 1A) supporting the administration of the varicella vaccine (VARIVAX® 0.5 ml, subcutaneous route), which contains a minimum of 1,350 PFU (Plaque-Forming Units) of varicella virus from the Oka/Merck strain, without suspension of the current immunosuppressive therapy (12).

**Table 1A:** Pre-vaccination patient immunocompetence assessment.

Laboratory test and immunocompetence criteria^a^	Patient results
Leucocytes (≥3,000/mm³)	15,050/mm³
Lymphocytes (≥1,200/mm³)	2,920/mm³
IgG (≥500 mg/dl)	621 mg/dl
CD4 + cell count (>200/mm³)	731/mm³
IgM (≥20 mg/dl)	183 mg/dl
Tetanus toxoid antibody (≥0.1 IU/ml)	1.83 IU/ml
Interferon Gamma Release Tuberculosis Assay (IGRA)	Negative
VZV-IgG (<200 mIU/ml)	<10 mIU/ml

^a^ According to the checklist regarding clinical and immunological requirements prior to varicella-zoster virus vaccination by Speth et al. (14).

**Figure 2:**
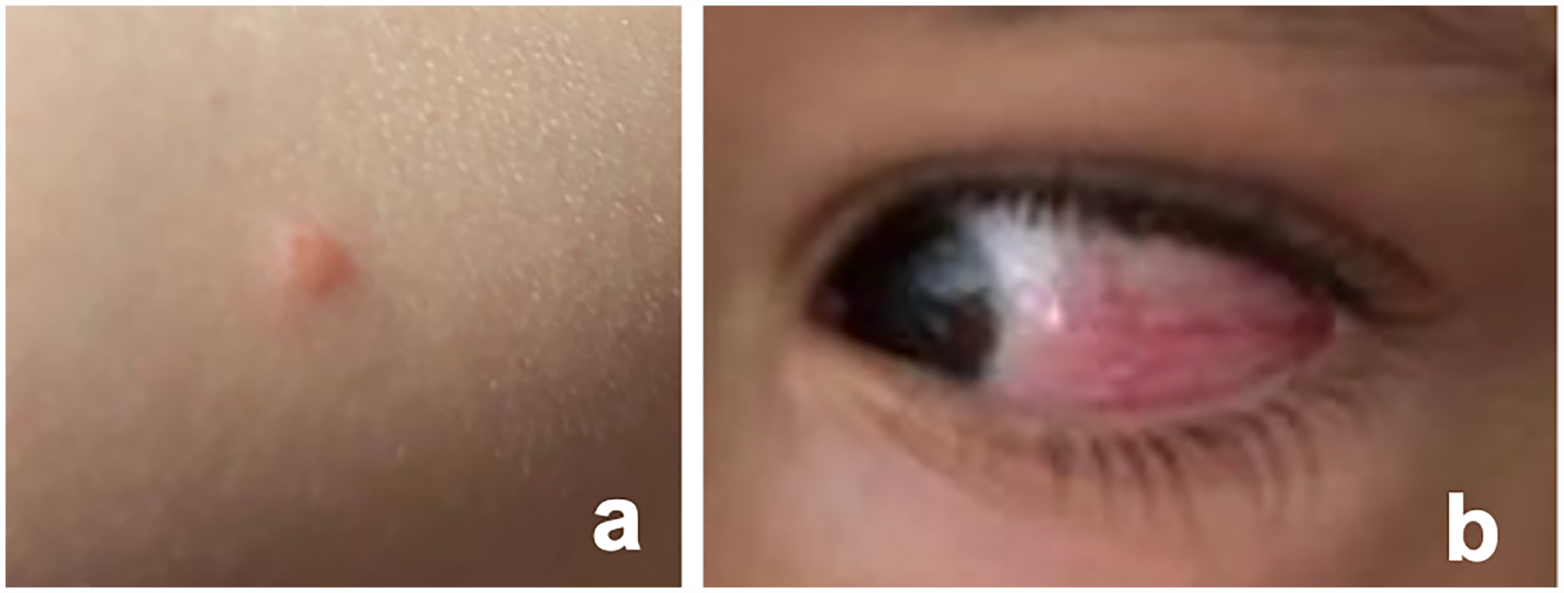
Vesicular lesion and ocular hyperemia post-vaccination. **(a)** Photographic documentation of one of the vesicular lesions that emerged 14 days post-vaccination on the right upper limb, the site where the vaccine was administered; **(b)** Evidence of left ocular hyperemia documented 16 days post-vaccination.

**Figure 3:**
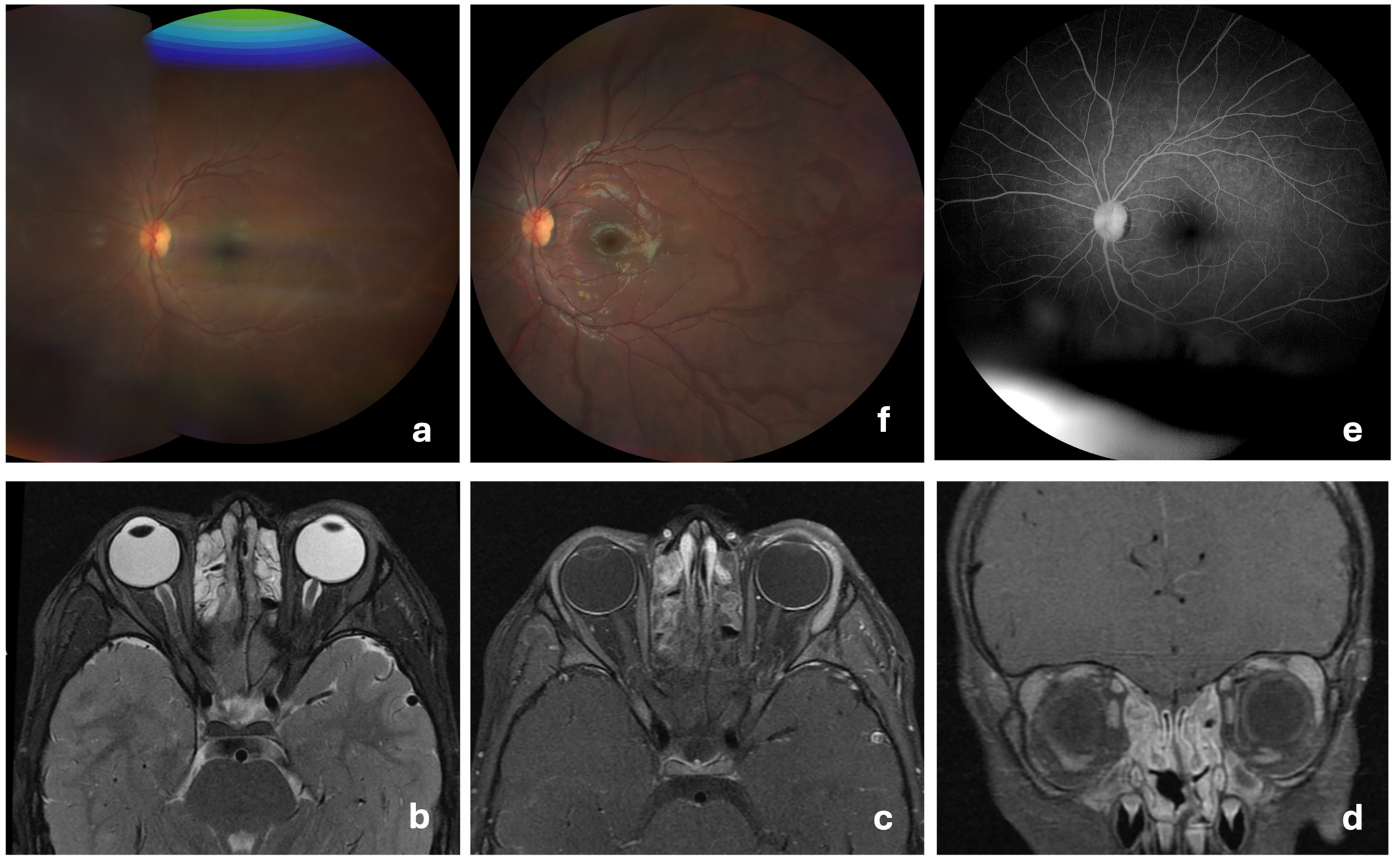
Retinal vasculitis and MRI findings at admission with follow-Up resolution. **(a)** At admission, fundus image of the left eye showing retinal arterial vasculitis, characterized by vessel wall irregularities, attenuation of the arteries, and signs of perivascular exudation; **(b–d)** Orbital MRI at admission shows enhanced contrast uptake in the left uveal tract (iris, ciliary body, and choroid) with mild anterior prominence at the optic disc. Anterior globe margin enhancement suggests conjunctival inflammation. Increased volume and contrast uptake in the left lacrimal gland indicate adenitis. Evidence of rhinosinusitis; **(e)** Two months post-discharge reassessment of the left eye, showing no evidence of sequelae on fundoscopic examination; **(f)** Two months post-discharge reassessment, with angiography documenting the resolution of vasculitis.

Fourteen days post-vaccination, three vesicular cutaneous lesions appeared on the right upper limb (Figure 2a), where the vaccine was administered. A swab was taken from the lesions, and varicella-zoster virus was detected via polymerase chain reaction (PCR). Two days after (day 16 post-vaccination), the child developed hyperemia in the left eye (Figure 2b), without pain, foreign body sensation, floaters, changes in visual acuity, or photophobia. He was evaluated at an emergency department without the knowledge of the team responsible for the vaccination decision, and after observation with no specific tests performed, a conjunctivitis was assumed. For this first event a course of topical antibiotic and topical corticosteroids was started, with no improvement. One month after vaccination, due to persistent ocular hyperemia in the absence of neurological or systemic involvement, the child was assessed by an ophthalmology team for the first time and was diagnosed with panuveitis in the left eye: numerous cells and a moderately severe flare in the anterior chamber, synechiae, papillitis, macular edema, fine keratic precipitates, and iris atrophy, associated with pronounced retinal arterial vasculitis (Figure 3a). The vaccination team was contacted and considering the possibility of post-varicella vaccine ophthalmic complications he was transferred to our unit for further investigation and treatment. An aqueous humor tap was performed along with a cranial and orbital MRI that corroborated findings consistent with panuveitis associated with mild papillary prominence and further disclosed associated discrete homolateral dacryoadenitis (Figures 3b–d). Analytically, C-reactive protein and erythrocyte sedimentation rate were within the reference range in the same fluid. From the etiological study (Table 1B), the notable finding was a positive PCR for VZV in the blood. However, sequencing to confirm the vaccine strain was not possible due to technical issues. Molecular testing for VZV in the aqueous humor could not be performed due to an insufficient sample volume.

**Table 1B:** Etiological study of panuveitis.

Infectious Causes		
Laboratory Test	Patient results	Reference levels
PCR for Varicella-Zoster Virus	Detected	-
HbsAg	Negative	–
HBsAb	102.0 IU/L	19 IU/L—immune
Anti-HCV	Negative	–
Anti-HIV 1/2	Negative	–
VDRL	Negative	–
Anti-CMV		
IgG	78 U/ml	>1.0 U/ml—positive
IgM	0.3 U/ml	>1.0 U/ml—positive
Anti-EBV		
VCA IgG	<10 U/ml	>25 U/ml—positive
VCA IgM	<10 U/ml	>40 U/ml—positive
EA IgG	<5 U/ml	>40 U/ml—positive
EBNA IgG	<3 U/ml	>40 U/ml—positive
Anti-Toxoplasma gondii		
IgG	1.2 IU/ml	>3.0 IU/ml—positive
IgM	Negative	
Anti-Bartonella		
IgG	<128	>128—positive
IgM	<32	>32—positive
Anti-Borrelia burgdorferi		
IgG	<5 UA/ml	>15 UA/ml—positive
IgM	6 UA/ml	>22 UA/ml—positive
Rose Bengal Test	Negative	–
IGRA QuantiFERON	Negative	
Immunological study		
IgG	9,64 g/L	5.93–17.30 g/L
IgA	1.32 g/L	0.33–3.60 g/L
IgM	0.97 g/L	0.55–2.1 g/L
C3	1,23 g/L	0.90–1.80 g/L
C4	0,28 g/L	0.10–0.40 g/L
Lupus anticoagulant	1.0	<1.2
Anti-Beta2 Glycoprotein 1		
IgG	<6.4 UQ	<20 UQ
IgM	2.4 UQ	<20 UQ
Anti-cardiolipin		
IgG	<2.6 UQ	<20 UQ
IgM	3.4 UQ	<20 UQ
ANA	Negative	–
Anti-dsDNA	<10 IU/ml	>40 IU/ml—positive
Anti-neutrophil cytoplasmic antibodies targeting proteinase 3	<2.3 UQ	<20 UQ
Anti-neutrophil cytoplasmic antibodies targeting myeloperoxidase	<3.2 UQ	<20 UQ
Rheumatoid Factor	<9 IU/ml	<14 IU/ml
HLA B27	Negative	
Other Parameters		
CRP	<0.6 mg/L	<5.0 mg/L
ESR	7 mm/h	<11 mm/h
AST	26 U/L	<41 U/L
ALT	14 U/L	<19 U/L
Total serum calcium	10.3 mg/dl	8.80–10.80 mg/dl
Urine calcium/creatinine ratio	0.2 mg/mg	<0.2 mg/mg
ACE	144 U/L	8–52 U/L
Lysozyme	992 ng/ml	700–2,580 ng/ml

ACE, angiotensin-converting enzyme; ALT, alanine aminotransferase; ANA, antinuclear antibodies; anti-CMV, cytomegalovirus antibody; anti-dsDNA, anti-double stranded DNA antibodies; anti-EBV, epstein-barr virus antibody; anti-HCV, total hepatitis C antibody; anti-HIV ½, HIV 1 + 2 antibody; ASO, anti-streptolysin O; AST, aspartate aminotransferase; C3, complement component 3; C4, complement component 4; CRP, C-reactive protein; EA, early antigen; EBNA, epstein-barr nuclear antigen; ESR, erythrocyte sedimentation rate; HBsAb, hepatitis B surface antibody; HBsAg, hepatitis B surface antigen; IGRA, interferon gama release assay; PCR, polymerase chain reaction; VCA, viral capsid antigen; VDRL, venereal disease research laboratory test for syphilis.

Nevertheless, given the clinical presentation, the temporal association with the varicella vaccination, the detection of VZV in the blood, and cutaneous lesions, post-vaccination ocular varicella was assumed even without recent varicella exposure. MMF treatment was discontinued. He completed 15 days of intravenous acyclovir (10 mg/kg/dose tid) and 15 days of systemic corticosteroids, including three pulses of methylprednisolone (30 mg/kg/dose), followed by prednisolone (1 mg/kg/day). Ophthalmologic treatment included prednisolone eye drops and ointment, along with cyclopentolate hydrochloride. The child showed favorable clinical progression, with improvement in ocular inflammation observed from day three of hospitalization. PCR for VZV in the blood became undetectable starting on day five of hospitalization. There were no signs of nephrotic syndrome relapse.

The patient was discharged on day 16 of hospitalization (day 46 post-vaccination), with outpatient therapy of therapeutic-dose valacyclovir (20 mg/kg/dose tid) for 6 days (totaling 21 days of antiviral therapy) followed by prophylactic dosing (20 mg/kg/dose bid). He completed an additional 10 days of oral prednisolone with gradual tapering (total of 25 days of systemic corticosteroid therapy). On day five post-discharge, MMF was reinitiated after two negative PCR tests for VZV. At the ophthalmology follow-up, two months post-discharge, he was clinically stable with no signs of sequelae (Figures 3e,f).

## Discussion

This case illustrates the complexity of interpreting immune status in children treated with immunosuppressive therapy and the side effects of using a live varicella vaccine in this population, even if immunocompetence criteria is met. Patients with nephrotic syndrome, due to the immunosuppression associated with both the disease's underlying pathophysiology, require a personalized vaccination schedule (16).

MMF active component, mycophenolic acid, inhibits inosine monophosphate dehydrogenase, a key enzyme in the *de novo* synthesis of guanosine nucleotides, thereby limiting the proliferation of T and B lymphocytes (17, 18). Effective cellular immunity is essential for halting VZV viremia, controlling its cutaneous replication, and preventing reactivation (19). Therefore, the immunosuppressive effects of MMF on lymphocyte proliferation may explain the increased susceptibility to VZV complications in patients undergoing treatment with this drug. According to the European Alliance of Associations for Rheumatology (EULAR), a patient on MMF is considered immunosuppressed if the dose is ≥30 mg/kg/day or >1,000 md/day (16). It is widely accepted that the history of VZV infection and vaccination should be documented in pediatric patients scheduled to receive high-dose disease-modifying antirheumatic drugs (DMARDs). If the patient is seronegative for VZV, vaccination should be given at least 2–4 weeks prior to the initiation of immunosuppressive therapy (20). According to the 2021 EULAR/PRES guidelines, administering the varicella vaccine to immunosuppressed patients is considered safe under certain conditions and is now strongly recommended for VZV-naïve patients receiving methotrexate and may also be considered for those on TNF inhibitors, anti-IL1, anti-IL6 therapies, or low-dose glucocorticosteroids. However, these guidelines do not specifically address other immunosuppressive drugs, such as MMF (16).

The literature reports viremia in 50% of healthy children who develop seroconversion following varicella vaccination (21). It is likely that these rates are even higher and/or more prolonged in immunosuppressed individuals, considering that in wild-type infection, viral shedding is more prolonged in immunocompromised hosts (22). The literature suggests that the vaccine virus follows an incubation period comparable to wild-type varicella, typically lasting from 7 to 21 days, with the peak occurrence around 14 days post-vaccination (23). Оur case had a scant vesicular rash, appearing 14 days post-vaccination without progression. Similarly, in Speth et al.'s (14) study, participants received written instructions to initiate acyclovir treatment if VZV disease presented with more than 50 skin lesions or if a rash lasted for more than seven days. They were also advised to contact their pediatric rheumatologist to discuss any potential need for reducing immunosuppressive therapy. However, our patient initially had far less severe clinical manifestations. The mere temporal association between an adverse event and the administration of the varicella vaccine does not necessarily imply that the vaccine is the direct cause of the event. Other possible infections, such as those caused by wild-type VZV or unrelated viruses, should be carefully excluded. The rash caused by wild-type varicella virus is classically described as having a cephalocaudal progression, beginning in the scalp near the hairline, and predominantly affecting the trunk, head, and face (4). In contrast, the initial involvement of the upper limb in our patient is less suggestive of wild-type virus infection. Moreover, our patient was previously immunized with MMR vaccination, known to cause pos-vaccination optic neuritis, although more than seven weeks occurred after inoculation, turning MMR a less probable cause (24). To accurately determine the cause, it is crucial to perform laboratory tests, such as on vesicular fluid or skin swabs, to confirm whether VZV is present and, if so, to identify whether the strain is of vaccine origin or wild-type VZV (25). Unfortunately, it was not possible to obtain sequencing of the detected VZV virus in our case. Additionally, the timeline of ophthalmologic manifestations is consistent, with the literature describing an interval of 1–3 weeks after vaccination, which aligns with a typical latency period for immune-triggered mechanisms (24).

A previously documented case of uveitis associated with varicella virus vaccine involved a 16-year-old girl who was otherwise healthy, that developed a generalized vesicular rash and ocular manifestations two days and seven post-vaccination, respectively (26). In our patient, uveitis symptoms appeared 16 days post-vaccination, but were milder, which may have contributed to a delay in the diagnosis of uveitis. A very recent review focusing on serious adverse events after live varicella/zoster vaccination suggests a potential association between the administration of the varicella vaccine in the upper limb and the underlying pathophysiological mechanisms implicated in cases of acute retinal necrosis, an association further supported by the absence of reported cases in children immunized via thigh injection (27). Proximal viremia—closer to the head—could allow the virus to reach the retina directly through the central retinal artery, a branch of the ophthalmic artery, which in turn arises from the internal carotid artery (27). The viremia detected in our patient, who was immunized in the upper limb, along with the identification of retinal arterial vasculitis on ophthalmologic examination at admission, supports this proposed mechanism. These observations raise the question of whether varicella vaccination in the upper limb should be avoided in young children.

Uveitis can generally be categorized into two main groups based on its etiology: autoimmune/inflammatory causes or infectious origins. The detection of specific intraocular antibodies, as calculated by a Goldmann-Witmer coefficient of 3 or higher, and/or the identification of viral DNA via PCR in aqueous humor, is considered definitive evidence of a viral etiology (15, 28).

The disease course may be uniphasic or chronic relapsing. Long-term maintenance with oral antivirals may help in minimizing recurrences, although there is no consensus (15). Macular edema is a common cause of vision loss in uveitis patients and was noted in our case. Systemic corticosteroids are highly effective for controlling macular edema, often given in pulses followed by tapering (29).

The main limitation in this case was the inability to confirm VZV in the aqueous humor, as well as the failure to verify the strain's compatibility with the vaccine. The initial lack of awareness by the vaccination team regarding ocular symptoms may have delayed the recognition of their association with the vaccine.

This case underscores the importance of ongoing surveillance and reporting, along with prospective research in larger patient cohorts, to further clarify the safety, methods of vaccination, including vaccination site and efficacy of live attenuated vaccines in immunosuppressed patients.

## Data Availability

The original contributions presented in the study are included in the article/Supplementary Material, further inquiries can be directed to the corresponding author.
